# Relationship between nocturia and hypertension: findings from the NHANES 2005–2016

**DOI:** 10.3389/fcvm.2023.1165092

**Published:** 2023-07-06

**Authors:** Junhao Chen, Zhenghuan Liu, Luchen Yang, Jing Zhou, Kai Ma, Zhufeng Peng, Qiang Dong

**Affiliations:** ^1^ Department of Urology, Institute of Urology, West China Hospital, Sichuan University, Chengdu, China; ^2^ West China School of Medicine, Sichuan University, Chengdu, China

**Keywords:** nocturia, hypertension, epidemiology, clinical study, NHANES

## Abstract

**Objectives:**

The objective of this study was to explore the association between nocturia and hypertension in a large, nationally representative adult sample.

**Methods:**

We used data from 2005 to 2016 National Health and Nutritional Examination Surveys (NHANES). A total of 29,505 participants aged 20 years old or older were included. A participant was considered to have nocturia if he or she had two or more voiding episodes at night. Multivariable logistic regression models were used to explore the association between nocturia and hypertension.

**Results:**

Participants with nocturia were associated with a higher risk of hypertension (OR, 1.36; 95% CI, 1.28–1.45). Interaction tests revealed no significant effect of sex, age, race, or body mass index on the association of nocturia with hypertension. As the severity of nocturia increases, the risk of hypertension increases (*P* for trend <0.0001). In addition, nocturia was also related to different grades of hypertension (II vs. I: OR, 1.34, 95% CI, 1.16–1.55; III vs. I: OR, 1.67, 95% CI, 1.32–2.13).

**Conclusion:**

In this cross-sectional study, our results suggest that nocturia is associated with an increased risk for hypertension.

## 1. Introduction

Nocturia was defined as a person who had one or more interruptions in sleep due to the need to urinate (1). The definition is currently controversial. One voiding episode at night is common, and two or more voiding episodes nightly were considered to be more clinically meaningful. Nocturia is a clinical manifestation of lower urinary tract symptoms and can result in sleep loss, which is related to daytime sleepiness and a decline in quality of life. According to the data from the 2005–2012 NHANES, the prevalence of ≥2 voids/night was approximately 34.9% in women and 30.5% in men (2). In addition, the prevalence of ≥1 void/night increases with age in men (20–39 years, 56.8%; 40–59 years, 70.2%; ≥60 years, 82.7%) and women (20–39 years, 68.9%; 40–59 years, 74.3%; ≥60 years, 84.7%) (3). Although at least one urination overnight is sufficient to define nocturia, two or more voidings at night have been reported to be associated with more adverse clinical outcomes (4). A cross-sectional study including 14,114 participants reported that nocturia (≥2 voids/night) was related to cardiovascular disease (CVD), and the risk of CVD increased with increasing frequency of voiding at night (5). Among them, participants who urinated more than four times per night had a 74% increased risk of CVD (5). Another study enrolled 15,988 subjects aged 20 years old or older and reported that nocturia (≥2 voids/night) was a predictor of mortality and that the mortality risk increased with the increased number of voiding episodes nightly (6). Dutoglu et al. reported that ≥2 voids at night was related to insomnia, polypharmacy, decreased walking speed and recurrent falls for older women (4). The occurrence of nocturia is a condition with a variety of factors, including drinking too much water at night, decreased bladder capacity, increased postvoid residual volume, overactive bladder, sleep disorders, hypertension, diabetes and medications (e.g., diuretics, calcium channel blockers) (7, 8). Appropriate lifestyle interventions and treatment of underlying diseases are sufficient to reduce the incidence of nocturia to some extent.

Hypertension is a medical condition that significantly increases the risk of heart, brain, kidney and other diseases. According to WHO statistics, as of 2019, there were approximately 1.28 billion people aged 30–79 years with high blood pressure worldwide, which is 2 times that of 1990. High blood pressure is related to a large burden of cardiovascular disease and premature death. In 2015, approximately 7.8 million deaths were related to systolic blood pressure ≥140 mmHg globally, which was mainly attributed to ischemic heart disease, ischemic stroke and hemorrhagic stroke (9). There are also multiple risk factors for hypertension, including high sodium intake, low potassium intake, smoking, alcohol consumption, psychological stress, sleep disorders, obesity, unhealthy diet, and noise exposure (10). If the risk of hypertension is reduced, it will help reduce the global economic burden.

The relationship between nocturia and hypertension has been reported previously. Rahman et al. conducted a systematic review and meta-analysis including 25 studies to explore the association between nocturia and hypertension. The results revealed that ≥1 voiding episode at night was related to a 1.2-fold higher risk of hypertension, while ≥2 voiding episodes at night were associated with a 1.3-fold increased risk of hypertension (11). A cross-sectional analysis of data from a prospective national cohort enrolled 22,674 obstructive sleep apnea (OSA) patients, including 11,332 participants with hypertension and 11,342 subjects without hypertension. The results reported that OSA patients with nocturia (≥1 void/night) were more likely to have hypertension and that the risk of hypertension increased with increasing nocturia severity for OSA patients with 2 or more voiding episodes at night (12).

These findings suggest different degrees of an association between nocturia and hypertension. However, there is a paucity of cross-sectional studies with large samples of natural populations on this potential association that avoided confounding factors. Moreover, no study has explored the association between nocturia and different degrees of hypertension. Therefore, we conducted this study to comprehensively explore the relationship between nocturia and hypertension in adults 20 years old or older among the large population of the United States.

## 2. Methods

### 2.1. Data and population

The National Health and Nutrition Examination Survey (NHANES) is a nationally representative cross-sectional survey conducted in the United States to assess the health and nutrition status of adults and children. The data of our study were obtained by consolidating demographic data, examination data, laboratory data and questionnaire data from NHANES 2005–2016, which can be downloaded from its website (https://www.cdc.gov/nchs/nhanes/index.htm). The Research Ethics Review Board at the National Center for Health Statistics (NCHS) approved the survey protocol.

In total, 60,936 participants were investigated in the NHANES 2005–2016. We excluded participants who were under 20 years old (*N* = 26,756). Participants without questionnaire information about nocturia (*N* = 4,633) and hypertension (*N* = 42) were also excluded. Finally, 29,505 participants aged 20 years old or older with complete information about nocturia, hypertension and model covariates were enrolled in this study.

### 2.2. Exposure and outcome

Whether the participants had nocturia was assessed by using a questionnaire. “During the past 30 days, how many times per night did you most typically get up to urinate from the time going to bed at night to waking up in the morning?” The responses were divided into 0 void/night, 1 void/night, 2 voids/night, 3 voids/night, 4 voids/night and 5 voids/night. Participants with ≥2 voids/night were regarded as having nocturia in our study.

Whether the participants had high blood pressure was assessed in several ways. First, if a person responded “yes” to the question “Have you ever been told by a doctor or other health professional that you have hypertension or high blood pressure?”, he was considered to have hypertension. Second, if a participant self-reported using an antihypertensive drug, he was regarded as having hypertension. Finally, if a participant had systolic blood pressure ≥140 mmHg and/or diastolic blood pressure ≥90 mmHg in the physical examination, he was also regarded as a patient with hypertension. When data were missing for one assessment method, we used another method, and a participant who met any one of the above three methods was considered to have high blood pressure.

Among 29,505 participants, 26,050 subjects underwent 3 blood pressure measurements. We took the average of the results of the 3 blood pressure measurements as the participants' blood pressure value, according to which the participants' blood pressure status were divided into 4 levels (nonhypertension: systolic pressure <140 mmHg and diastolic pressure <90 mmHg; hypertension grade I: systolic pressure 140 mmHg–159 mmHg and/or diastolic pressure 90 mmHg–99 mmHg; hypertension grade II: systolic pressure 160 mmHg–179 mmHg and/or diastolic pressure 100 mmHg–109 mmHg; hypertension grade III: systolic pressure ≥180 mmHg and/or diastolic pressure ≥110 mmHg). Among them, there were 21,386 participants without hypertension, 3,451 participants with hypertension grade I, 922 participants with hypertension grade II and 291 participants with hypertension grade III.

### 2.3. Covariates

Based on previous studies, potential confounding variables that may affect the association between nocturia and hypertension were included in the multivariable models. Those covariates included gender, age, race, education level, marital status, family poverty income ratio (PIR), body mass index (BMI), smoking, alcohol consumption, diabetes, depression, cardiovascular disease (CVD), cancer, cholesterol and triglycerides level. The race of participants was divided into 4 categories, including Non-Hispanic white people, Non-Hispanic black people, Mexican American and others. Age (20–39, 40–59, ≥60), education level (less than high school, high school or equivalent and college or above), marital status (married, separated and never married), family PIR (<1.3, 1.3–3.5, >3.5), BMI (<25 kg/m^2^, 25–29.9 kg/m^2^, ≥30 kg/m^2^), smoking (never smoker, former smoker, current smoker), and alcohol consumption (nondrinker, low to moderate drinker, heavy drinker) were divided into 3 categories. Diabetes, depression, CVD (congestive heart failure, coronary heart disease, heart disease, angina pectoris or stroke) and cancer status were divided into 2 categories. Depression was assessed by using the Nine-item Patient Health Questionnaire (PHQ-9) depression scale, and a PHQ-9 score ≥10 was considered to indicate depression. Cholesterol and triglyceride levels were regarded as continuous variables.

### 2.4. Statistical analysis

For the baseline characteristics, categorical variables are presented as the number of cases and percentage, while continuous variables are presented as the mean ± standard deviation. The baseline characteristics of participants were categorized by hypertension status, and the differences between the two groups were calculated by the chi-square test for categorical variables and the t test for continuous variables. A multivariable logistic regression model was used to explore the association between nocturia and hypertension. The degree of association was represented by the odds ratio (OR) and 95% confidence interval (CI). Multivariable models included the nonadjusted model 1, minimally adjusted model 2 (sex, age and race were adjusted), and fully adjusted model 3 (covariates of model 2 combined with education level, marital status, family PIR, BMI, smoking status, alcohol consumption, diabetes, depression, CVD, cancer, cholesterol and triglycerides level were adjusted). Interaction and stratified analyses were performed according to sex, age (20–39, 40–59, ≥60), race (Non-Hispanic white people, Non-Hispanic black people, Mexican American and others), and BMI (<25 kg/m^2^, 25–29.9 kg/m^2^, ≥30 kg/m^2^). Multinomial logistic regression was conducted between nocturia and different degrees of hypertension. The software packages R (http://www.R-project.org, The R Foundation) and Empowerstats (http://www.empowerstats.com) were used to perform all statistical analyses. *P* < 0.05 was regarded as statistically significant.

## 3. Results

### 3.1. Demographic characteristics

The baseline characteristics of the study participants based on blood pressure status are presented in Table 1. A total of 29,505 participants aged 20 years or older, including 14,501 males and 15,004 females, were enrolled in the study. The prevalence of hypertension and nonhypertension was 36.11% and 63.89%, respectively. Participants with hypertension were more likely to be female, ≥60 years old, non-Hispanic white, married, and have less education, lower family PIR, higher BMI, higher level of triglycerides, more alcohol consumption and smoking. In addition, the hypertension group was more likely to have diabetes, depression, CVD, and cancer.

**Table 1:** Characteristics of participants in national health and nutritional examination surveys 2005–2016 (Chengdu, China. 2022).

	Non-hypertension *N* = 18,850	Hypertension *N* = 10,655	*P*-value
Cholesterol (mmol/L)	5.06 ± 1.05	4.98 ± 1.11	<0.001
Triglycerides (mmol/L)	1.68 ± 1.49	1.92 ± 1.44	<0.001
Gender			0.005
Male	9,381 (49.77%)	5,120 (48.05%)	
Female	9,469 (50.23%)	5,535 (51.95%)	
Age (year)			<0.001
20–39	8,802 (46.69%)	1,200 (11.26%)	
40–59	6,237 (33.09%)	3,345 (31.39%)	
≥60	3,811 (20.22%)	6,110 (57.34%)	
Race			<0.001
Non-Hispanic white people	8,147 (43.22%)	4,849 (45.51%)	
Non-Hispanic black people	3,398 (18.03%)	2,852 (26.77%)	
Mexican American	3,427 (18.18%)	1,291 (12.12%)	
Others	3,878 (20.57%)	1,663 (15.61%)	
Education level			<0.001
Less than high school	4,432 (23.51%)	3,051 (28.63%)	
High school or equivalent	4,158 (22.06%)	2,598 (24.38%)	
College or above	10,246 (54.36%)	4,994 (46.87%)	
Missing	14 (0.07%)	12 (0.11%)	
Marital status			<0.001
Married	12,221 (64.83%)	8,638 (81.07%)	
Separated	578 (3.07%)	400 (3.75%)	
Never married	6,040 (32.04%)	1,613 (15.14%)	
Missing	11 (0.06%)	4 (0.04%)	
Family PIR			<0.001
<1.3	5,362 (28.45%)	3,213 (30.15%)	
1.3–3.5	7,896 (41.89%)	4,675 (43.88%)	
>3.5	5,592 (29.67%)	2,767 (25.97%)	
BMI (kg/m^2^)			<0.001
<25	6,546 (34.73%)	1,905 (17.88%)	
25–29.9	6,517 (34.57%)	3,519 (33.03%)	
≥30	5,787 (30.70%)	5,231 (49.09%)	
Smoking status			<0.001
Never smokers	10,803 (57.31%)	5,276 (49.52%)	
Former smokers	3,898 (20.68%)	3,350 (31.44%)	
Current smokers	4,137 (21.95%)	2,020 (18.96%)	
Missing	12 (0.06%)	9 (0.08%)	
Alcohol consumption			<0.001
Non-drinkers	5,059 (26.84%)	3,467 (32.54%)	
Low to moderate drinkers	12,127 (64.33%)	6,320 (59.31%)	
Heavy drinkers	1,664 (8.83%)	868 (8.15%)	
Diabetes			<0.001
Yes	1,109 (5.88%)	2,645 (24.82%)	
No	17,731 (94.06%)	8,001 (75.09%)	
Missing	10 (0.05%)	9 (0.08%)	
Depression			<0.001
Yes	1,335 (7.08%)	1,224 (11.49%)	
No	17,515 (92.92%)	9,431 (88.51%)	
Severity of nocturia (Voids/night)			<0.001
0	7,204 (38.22%)	2,161 (20.28%)	
1	7,064 (37.47%)	3,687 (34.60%)	
2	2,853 (15.14%)	2,574 (24.16%)	
3	1,105 (5.86%)	1,419 (13.32%)	
4	368 (1.95%)	469 (4.40%)	
5	256 (1.36%)	345 (3.24%)	
Nocturia			<0.001
Yes	11,646 (61.78%)	8,494 (79.72%)	
No	7,204 (38.22%)	2,161 (20.28%)	
CVD			<0.001
Yes	819 (4.34%)	2,405 (22.57%)	
No	17,988 (95.43%)	8,169 (76.67%)	
Missing	43 (0.23%)	81 (0.76%)	
Cancer			<0.001
Yes	1,187 (6.30%)	1,637 (15.36%)	
No	17,649 (93.63%)	9,007 (84.53%)	
Missing	14 (0.07%)	11 (0.10%)	

PIR, poverty income ratio; BMI, body mass index; CVD, cardiovascular disease; *χ*^2^ test for categorical variables. *T*-test for continuous variables.

### 3.2. Association between nocturia and hypertension

The results of multivariable logistic regression analysis are presented in Table 2. Our study revealed that nocturia (≥2 voids/night) was associated with an increased risk of hypertension (Model 1, OR, 2.56, 95% CI, 2.43–2.69; Model 2, OR, 1.69, 95% CI, 1.60–1.79). After full adjustment, participants with nocturia had a 36% higher risk of hypertension (Model 3, OR, 1.36, 95% CI, 1.28–1.45). In addition, as the frequency of voiding episodes at night increases, so does the risk of hypertension. When no variables were adjusted in Model 1, the OR values of 1 void/night vs. 0 void/night, 2 voids/night vs. 0 void/night, 3 voids/night vs. 0 voids/night, 4 voids/night vs. 0 void/night, and ≥5 voids/night vs. 0 void/night were 1.74, 3.01, 4.28, 4.25, and 4.49, respectively. The association remained stable after adjusting for gender, age, and race in Model 2. Even though all covariables included were adjusted in Model 3, participants with 1 void/night, 2 voids/night, 3 voids/night, and 4 voids/night were associated with 22%, 45%, 68%, and 53% higher risks of hypertension, respectively, than those with 0 voids/night. The risk even increased to 83% for 5 voids/night vs. 0 voids/night. The *P*-value of trend testing was <0.0001 for all models, which further indicated that with the increase in the severity of nocturia, the risk of hypertension increases.

**Table 2:** Association between nocturia and hypertension among participants in national health and nutritional examination surveys 2005–2016 (Chengdu, China. 2022).

Exposure	Model 1 (OR, 95% CI)	Model 2 (OR, 95% CI)	Model 3 (OR, 95% CI)
**Nocturia**			
No	1.0	1.0	1.0
Yes	2.56 (2.43, 2.69)	1.69 (1.60, 1.79)	1.36 (1.28, 1.45)
**Severity of nocturia (Voids/night)**			
0	1.0	1.0	1.0
1	1.74 (1.63, 1.85)	1.28 (1.20, 1.37)	1.22 (1.13, 1.31)
2	3.01 (2.80, 3.23)	1.72 (1.59, 1.86)	1.45 (1.34, 1.58)
3	4.28 (3.90, 4.69)	2.26 (2.04, 2.50)	1.68 (1.51, 1.87)
4	4.25 (3.68, 4.91)	2.31 (1.97, 2.71)	1.53 (1.29, 1.82)
5	4.49 (3.80, 5.32)	2.88 (2.39, 3.46)	1.83 (1.50, 2.23)
*P* for trend	<0.0001	<0.0001	<0.0001

Model 1 was adjusted for none.

Model 2 was adjusted for gender, age, and race.

Model 3 was adjusted for Model 2, education level, marital status, PIR, BMI, smoking status, alcohol consumption, diabetes, depression, CVD, cancer, cholesterol, and triglycerides.

There was still a positive relationship between nocturia and the risk of hypertension when stratifying by sex, age, race and BMI (Table 3). No significant difference was found by the interactions testing the association between nocturia and hypertension among different groups of sex, age, race, and BMI (*P* for interaction = 0.16, 0.53, 0.58, 0.96), which indicated that the association was not affected by these baseline characteristics.

**Table 3:** Stratified analyses of association between nocturia and hypertension according to gender, age, race and BMI (Chengdu, China. 2022).

Subgroup	No. of subjects	OR, 95% CI	*P* for interaction
Gender			0.16
Male	14,501	1.33 (1.22–1.45)	
Female	15,004	1.45 (1.33–1.58)	
Age			0.53
20–39	10,002	1.45 (1.24–1.69)	
40–59	9,582	1.42 (1.29–1.58)	
≥60	9,921	1.28 (1.17–1.40)	
Race			0.58
Non-Hispanic white people	12,996	1.32 (1.20–1.44)	
Non-Hispanic black people	6,250	1.43 (1.26–1.62)	
Mexican American	4,718	1.40 (1.19–1.64)	
Others	5,541	1.33 (1.14–1.55)	
BMI (kg/m^2^)			0.96
<25	8,451	1.38 (1.21–1.58)	
25–29.9	10,036	1.35 (1.22–1.50)	
≥30	11,018	1.36 (1.24–1.49)	

Stratified analyses were adjusted for gender, age, race, education level, marital status, PIR, BMI, smoking status, alcohol consumption, diabetes, depression, CVD, cancer, cholesterol, and triglycerides. When a stratified analysis of an independent variable is performed, the independent variable itself is not adjusted.

To explore the relationship between nocturia and different degrees of hypertension, multinomial logistic regression was conducted between nocturia and hypertension grades I, II and III (Table 4). The results revealed that in participants with hypertension grade II, those with nocturia had a 34% higher risk of hypertension grade II than those with hypertension grade I (OR, 1.34, 95% CI, 1.16–1.55). In participants with hypertension grade III, those with nocturia had a 67% higher risk of hypertension grade III than those with hypertension grade I (OR, 1.67, 95% CI, 1.32–2.13).

**Table 4:** Multinomial logistic regression models evaluating the association of nocturia and different grades of hypertension (Chengdu, China. 2022).

Hypertension grades	Nocturia (Ref. Non-nocturia)		
	Subjects	OR, 95% CI	*P*-value
Hypertension I	3,451	1.0 (Ref.)	–
Hypertension II	922	1.34 (1.16, 1.55)	<0.001
Hypertension III	291	1.67 (1.32, 2.13)	<0.001

## 4. Discussion

In this study, we enrolled 29,505 participants aged 20 years or older in the US to explore the association between nocturia and hypertension. The results revealed that participants with nocturia (voiding ≥2/night) were more likely to have hypertension, and this association did not appear to be affected by sex, age, race or BMI. In addition, the higher the frequency of voiding episodes at night, the more severe the symptoms of nocturia, and the severity of nocturia was also positively associated with the risk of hypertension. Moreover, when hypertension was divided into different degrees, positive associations were also detected between nocturia and different hypertension grades. To our knowledge, this is the first cross-sectional study with a large national population to explore the association between nocturia and hypertension.

A population-based study in Finland including 6,000 subjects reported that nocturia was related to age and that the prevalence rates of nocturia increased at a constant rate with age (13). Because the incidence of prostatic hyperplasia is increased in men over 50 years of age, nocturia prevalence rises steeply after 50 years old. A multicountry sample survey including 73,607 participants showed that the incidence of hypertension increases with age (14). The mechanism underlying age-related hypertension has not been fully elucidated, but aging was reported to be associated with arterial stiffness, elevated sympathetic nervous system activity and renal sodium transport systems, which was closely related to the occurrence of hypertension (15, 16). Therefore, the above two points explain to some extent why the relationship between nocturia and hypertension was not related to age in our study.

A systematic review and meta-analysis conducted by Rahman et al. reported that the association between nocturia and a higher risk of hypertension was more robust in females than in males (11). The results of our study also revealed that there were 33% and 45% higher risks of hypertension in subjects with nocturia for males and females, respectively, although the difference was not statistically significant. Possibly because nocturia has been regarded for a long time as the main male symptom caused by BPH, studies on nocturia in female patients have rarely been reported (17). Further investigation is required to understand the mechanism of this relationship.

African Americans are known to have a higher prevalence of hypertension than white and they have a somewhat higher incidence of enuresis and nocturia. Previous studies have shown that the prevalence of nocturia and hypertension is ethnically linked, with higher rates, especially in non-Hispanic black people (3, 18, 19). A large community-based cross-sectional study conducted by Victor et al. tested the hypothesis that nocturia is a common symptom of black people with hypertension, which can be used as a screening tool for high blood pressure (20). Although non-Hispanic black people with nocturia had the highest risk of hypertension in our study, the difference among different races was not statistically significant. There is no evidence that racial disparities in the risk of hypertension and nocturia can be explained by genetic factors. Sociodemographic, environmental and behavioral factors are therefore likely to be the main contributors. Further studies are needed to explore the racial and ethnic differences in the association between nocturia and hypertension. The prevalence of nocturia and hypertension was both reported to increase with increasing body mass index (21, 22). However, the association between nocturia and hypertension was reported to be unrelated to BMI (11), which was consistent with our findings. This indicated that nocturia has the potential to be used as a screening tool to identify hypertension among people with different weights.

There are multiple causes of nocturia. In males with symptoms of BPH there seems to be at least 2 groups of nocturians. Those with a small functional bladder and those with a slightly higher blood pressure during daytime, an accumulation of sodium and then a nighttime natriuresis. The sleep apnoea is related to hypertension and increased BNP and decreased O2 tension during the apnoea leading to natriuresis and nocturia (23). Sleep deprivation leads to attenuation of the sleep dipping in BP and increased diuresis. Attenuated dipping is related to an attenuation of the increase in AVP in the beginning of the night, mostly in men. Sleep deprivation leads to attenuation of the sleep dipping in BP and increased diuresis. Attenuated dipping is related to an attenuation of the increase in AVP in the beginning of the night, mostly in men (24). With age, there is a shift from AVP increase during the night (first third) to BNP. About females, sequelae after delivery with urologic cystocele. They tend to restore some of the cystocele during night and then have to void.

There may be a bidirectional influence between nocturia and hypertension. Patients with hypertension tend to have symptoms of nocturnal urination, which is related to physiological and pathological changes caused by high blood pressure. Hypertension can result in nocturia by altering the glomerular filtration rate and tubular transport (25). Patients with hypertension may have symptoms of peripheral edema, which can lead to an increase in nocturia when reabsorbed at night. Congestive heart failure is one of the complications of hypertension and leads to increased atrial tone, resulting in the release of atrial natriuretic peptides, which increases urine output (26). Elevated systolic blood pressure was accompanied by a decrease in serum levels of the antidiuretic vasopressin at night, causing an increase in nocturia (27). However, there have been few studies on the mechanism of the effect of nocturia on hypertension. A systematic review and meta-analysis conducted by Wang et al. involving 85,838 subjects reported that short sleep was associated with a 16% higher risk of hypertension (28). In addition, poor sleep quality was also reported to be related to an increased risk of new-onset hypertension among people aged 18 years or older (29). However, having 2 or more voiding episodes at night could disturb sleep, resulting in a deterioration in sleep quality or a decrease in sleep duration, which is associated with an increase in nocturnal blood pressure and the development of hypertension during the daytime. On the one hand, short sleep can disturb circadian rhythmicity and autonomic nervous system balance, resulting in heightened sympathetic nervous system activity (30), which plays a role in the occurrence of elevated blood pressure. On the other hand, people who are sleep-deprived are in a state of stress, which can promote salt intake and reduce salt excretion from the kidney (31). However, excessive salt intake has been demonstrated to be associated with the risk of hypertension. Moreover, chronic sleep deprivation can increase the risk of obesity and diabetes by influencing glucose metabolism and promoting excessive food intake through neuroendocrine regulation (32), which has been proven to be a risk factor for high blood pressure.

This study had some limitations. First, the assessment of nocturia and hypertension for participants was based on self-questionnaires, which were subjective and not entirely accurate. Second, the grade of hypertension was defined by the results of blood pressure measurement; however, some participants may have taken antihypertensive drugs, which will have an impact on the results. Third, the study is a cross-sectional design, which can only explore the relevant associations and cannot infer causal relationships.

## 5. Conclusion

Nocturia was found to be associated with an increased risk of hypertension. As the severity of nocturia increases, the risk of hypertension increases. Thus, it is necessary to take appropriate measures to alleviate the symptoms of nocturia, thereby reducing the risk of hypertension. For example, the treatment of prostatic hyperplasia, sleep disorders and other diseases that can easily lead to nocturia. For patients with high blood pressure, we should ask whether they have symptoms of nocturia to provide more precise prevention and treatment. Further studies are also needed to investigate its mechanism.

## Data Availability

The original contributions presented in the study are included in the article, further inquiries can be directed to the corresponding author.
